# Addition of Synthetic Biomaterials to Deproteinized Bovine Bone Mineral (DBBM) for Bone Augmentation—A Preclinical In Vivo Study

**DOI:** 10.3390/ijms231810516

**Published:** 2022-09-10

**Authors:** Masako Fujioka-Kobayashi, Hiroki Katagiri, Niklaus P. Lang, Jean-Claude Imber, Benoit Schaller, Nikola Saulacic

**Affiliations:** 1 Department of Cranio-Maxillofacial Surgery, University Hospital, University of Bern, 3010 Bern, Switzerland; 2 Department of Oral and Maxillofacial Surgery, School of Life Dentistry at Tokyo, The Nippon Dental University, Chiyoda-ku, Tokyo 102-8159, Japan; 3 Advanced Research Center, The Nippon Dental University School of Life Dentistry at Niigata, 1-8 Hamauracho, Chuo-ku, Niigata 951-8580, Japan; 4 Department of Periodontology, School of Dental Medicine, University of Bern, 3010 Bern, Switzerland

**Keywords:** in vivo, biomaterials, tricalcium phosphate, biphasic calcium phosphate, bone augmentation

## Abstract

(1) Aim: To investigate the effect of synthetic bone substitutes, α-tricalcium phosphate (α-TCP) or bi-layered biphasic calcium-phosphate (BBCP) combined with deproteinized bovine bone mineral (DBBM), on bone formation. (2) Methods: Thirty critical size defects were randomly treated with the following five different treatment modalities: (1) negative control (NC, empty), (2) DBBM, (3) α-TCP + DBBM (1:1), (4) BBCP 3%HA/97%α-TCP + DBBM (1:1), and (5) BBCP 6%HA/94%α-TCP + DBBM (1:1). The samples, at four weeks post-surgery, were investigated by micro-CT and histological analysis. (3) Results: A similar level of new bone formation was demonstrated in the DBBM with α-TCP bone substitute groups when compared to the negative control by histomorphometry. DBBM alone showed significantly lower new bone area than the negative control (*p* = 0.0252). In contrast to DBBM, the micro-CT analysis revealed resorption of the α-TCP + DBBM, BBCP 3%HA/97%α-TCP + DBBM and BBCP 6%HA/94%α-TCP + DBBM, as evidenced by a decrease of material density (*p* = 0.0083, *p* = 0.0050 and *p* = 0.0191, respectively), without changing their volume. (4) Conclusions: New bone formation was evident in all defects augmented with biomaterials, proving the osteoconductive properties of the tested material combinations. There was little impact of the HA coating degree on α-TCP in bone augmentation potential and material resorption for four weeks when mixed with DBBM.

## 1. Introduction

Bone augmentation techniques related to the placement of dental implants are common clinical procedures performed in oral and maxillofacial surgery. Hence, over 50% of dental implants are placed concomitantly with bone grafting procedures [1]. Guided bone regeneration (GBR) using biomaterials underneath a membrane is a highly predictable treatment to augment missing bone volume around implants, especially in the atrophic jaws [2]. After more than 20 years of experimental and clinical evidence, the use of bone graft substitutes has become a standard of care for bone augmentation [3]. In relation to the type of defect, the graft should possess appropriate features to allow its fixation in the recipient site and three-dimensional stability to withstand forces [4]. Hereby, autogenous bone grafts are considered a “standard”. However, the main drawback of utilizing such grafts, includes the need for a second surgical site and, therefore, donor site morbidity [5]. To avoid this, a granular form of bone substitutes (e.g., deproteinized bovine bone mineral, DBBM) has been widely used [6,7]. The DBBM is one of the most commonly employed bone substitutes composed of spongy bovine bone with trabecular structure and internal voids, completely free of organic components [8]. Bone grafts are biocompatible and preferably also osteoconductive, allowing new bone formation [9]. Despite these benefits, knowledge on the use of bone substitutes in the treatment of alveolar ridge atrophy is limited. In general, the choice of applied biomaterials, as well as the time of implant placement, have been derived from trial and error studies [10]. For example, healing times may be prolonged when compared to grafting protocols that include autogenous bone grafts in particulate form [11]. It is, therefore, of major clinical interest to elaborate on the healing process, including the course of degradation of particulate bone substitutes and the rate of new bone formation, with respect to the various compositions.

Several characteristics, such as the chemical composition, the granular size, the degree of hydrophilicity, the capillarity, the intra- and inter-granular porosity, and, in particular, the degradability, may have an impact on bone formation [12]. In that respect, it was demonstrated that synthetic degradable bi-layered biphasic calcium-phosphate (BBCP) comprised of a α-tricalcium phosphate (α-TCP) core coated with nanocrystalline biomimetic hydroxyapatite (HA) was biocompatible and osteoconductive leading to the healing of critical size defects in rabbits [13]. Since a rapid degradation might lead to an insufficient amount of newly formed mineralized bone [11], an assessment of a combined use with materials known for long-term volume maintenance is warranted. DBBM provides long term volume maintenance [14,15,16], being slowly resorbed by osteoclast cells [17]. The aim of the present preclinical study was: (i) to assess the impact of BBCP and α-TCP on de novo bone formation in combination with DBBM and (ii) to analyze the influence of HA coating degree on resorptive potential of α-TCP in combination with DBBM after four weeks. The null-hypothesis was that of no difference in the osteogenic potential of DBBM compared to biomaterials composed of a mixture of α-TCP or BBCP with the DBBM.

## 2. Results

**Figure 1:**
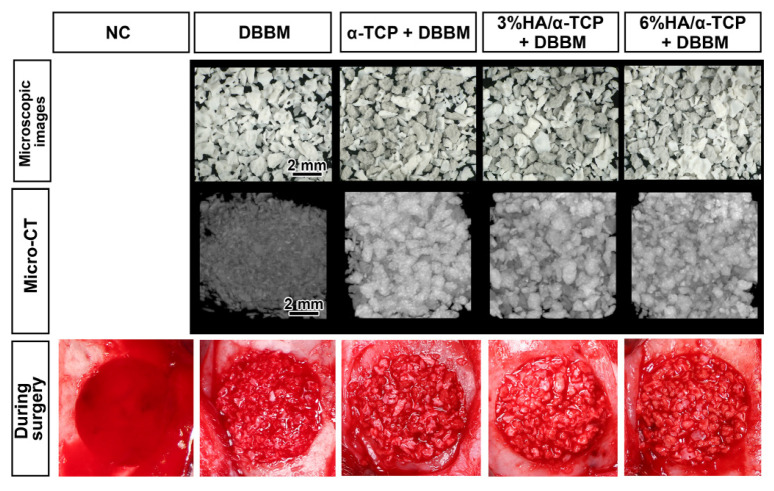
The images of the five tested groups: negative control (NC), DBBM alone, α-TCP + DBBM (1:1), 3%HA/97%α-TCP + DBBM (1:1), and 6%HA/94%α-TCP + DBBM (1:1). The microscopic images and micro-CT image of the substitutes before surgery and the implanted materials during surgery.

DBBM, the combination of DBBM with α-TCP or BBCP 3%HA/97%α-TCP or BBCP 6%HA97% α-TCP were of similar appearance, with the same size distribution of granules and a rough surface (Figure 1). During surgeries, all tested materials were easily soaked in blood, and well-handled in the defects.

Among 30 defects of a total of 15 treated animals, the data of one defect was removed due to the severe damage of the *dura mater* during surgery. No animals showed any signs of local wound dehiscence, exposure, inflammation, or infection at the surgical sites.

### 2.1. Micro-CT Analysis in Whole Defects

**Figure 2:**
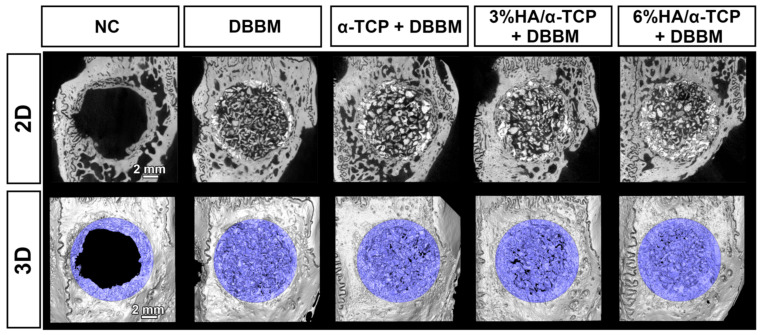
Micro-CT images of each group; 2D plane (**upper row**) and 3D-reconstructed views (**lower row**); the blue color shows mineralized tissue in the bone defects). Homogenously distributed mineralized structures comprising bone substitute materials and newly formed bone were observed in the defects. Scale bar: 2 mm.

**Figure 3:**
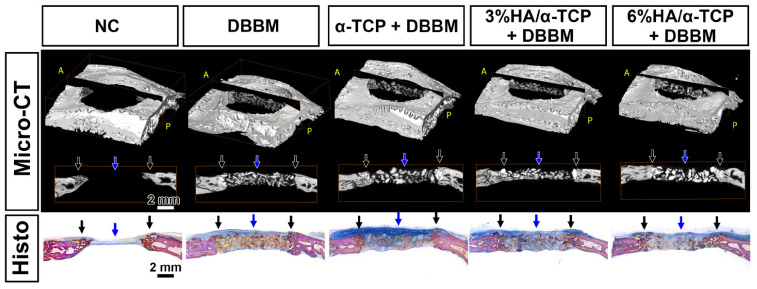
Sagittal sections of the micro-CT images and the corresponding toluidine blue and fuchsin staining at 4 weeks. All groups presented new bone in the peripheral areas (black arrows), and minor mineralized bone formation with abundant connective tissues surrounding to the materials were observed in the central areas of the defects (blue arrows). (Scale bars: 2 mm, A: Anterior, P: Posterior).

All the tested synthetic materials showed higher mineral density when compared to DBBM at 4 weeks analyzed in volume of interest (VOI_1, Table 1, Figure 2 and Figure 3). Micro-CT revealed homogenously distributed mineralized structures throughout the whole defect comprising bone substitute materials and newly formed bone. New mineralized bone had formed at the borders of all defects. However, newly formed bone could not be precisely distinguished from the residual bone substitute material because the material density was very similar among those, especially for DBBM (Figure 2 and Figure 3). Most of the implanted materials appeared non-resorbed after 4 weeks of healing. All material groups showed greater mineralized tissue volume/total tissue volume (MV/TV) and mineral density (MD) when compared to negative control (NC) group (Table 1). Furthermore, material groups could augment similar levels of MV/TV to reference intact bone (Table 1). A significantly higher mineral density in the defects at 4 weeks was observed in α-TCP + DBBM when compared to DBBM alone (*p* = 0.0319; Table 1). No significant MD differences could be shown for BBCP_3 or BBCP_6 in comparison to DBBM. The percentages of horizontal defect closure on a sagittal plane (HDC) with new bone were analyzed on the sagittal sections of the defects (Figure 3) and the similar levels of HDC were observed among groups (Table 1).

### 2.2. Histological Analysis

**Figure 4:**
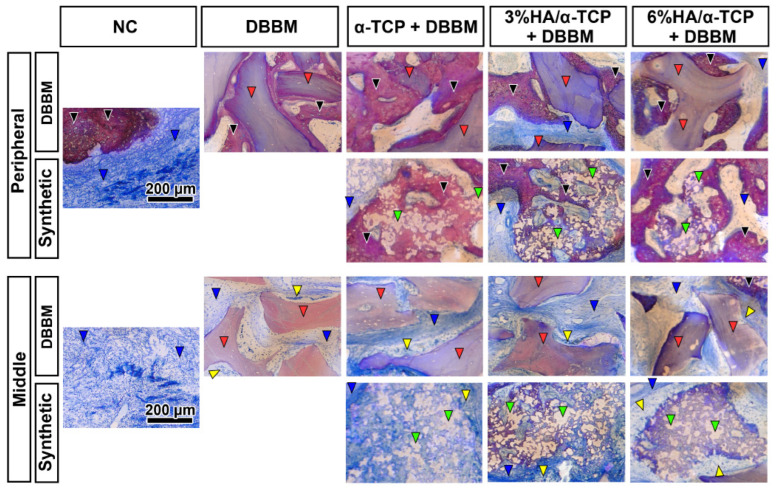
Magnified views of toluidine blue and fuchsin staining in the peripheral and central areas in each group at 4 weeks post-surgery. All groups showed great osteoconductive potential in the peripheral areas, whereas little bone was observed in the middle areas of the defects. New bone (black arrowheads) was observed on all tested synthetic biomaterials (green arrow heads) as well as DBBM (red arrow heads) in the peripheral area of the defects. In the central defect areas, multinucleated cells (yellow arrowheads) surrounded all types of the granules, embedded in the soft connective tissue (blue arrowheads). (Scale bars: 200 μm).

All groups presented new mineralized bone in the peripheral area (Figure 3 black arrows), and minor bone formation with abundant connective tissues surrounding the materials (Figure 3 blue arrows) were observed in the central area of the defects. The magnified histological views further confirmed the osteoconductive potential of all tested biomaterials, as well as DBBM in the peripheral area of the defects (Figure 4 black arrows). Appositional bone formation was observed in the case of the DBBM granules, whereas bone tissue infiltrated the granules of all different synthetic bone substitutes in a mesh like pattern reminiscent of resorption via creeping substitution. In the central defect area, multinucleated cells surrounded all types of the granules embedded in well vascularized soft connective tissue (Figure 4 blue arrows).

**Figure 5:**
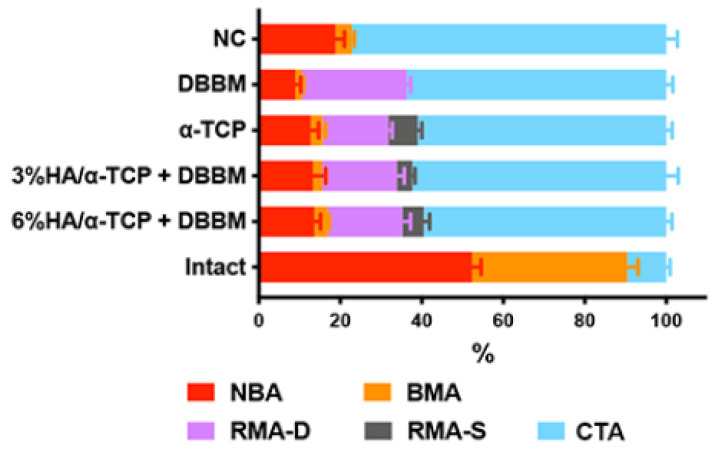
The summary of histomorphometry including NBA, BMA, RMA-D, RMA-S and CTA. The data of the intact calvarial bone without surgery is shown as a reference. The addition of the synthetic materials to DBBM increased the NBA to a level comparable to the NC group.

Histomorphological analysis revealed that none of the groups induced bone formation comparable to the reference, the intact bone (Table 2, Figure 5). However, the DBBM group showed less new mineralized bone area (NBA) than the NC (*p* = 0.0252), while the addition of the synthetic materials to DBBM increased the NBA to a level comparable to the NC. In line with the micro-CT analysis, the histomorphometry showed no significant differences in HDC among the tested groups (Table 2).

### 2.3. Material Resorption Assay by Micro-CT Analysis in the Central Area of the Defects

Histology revealed no significant differences in HDC despite the presence of osteoclast like cells, indicative of a resorption process in the center of the defects. Thus, the mineral density in the center of the defect, as an additional indicator for degradation, was measured. To this end, the change of the MD from day 0 (=initial material density of the material at implantation) to week 4 was calculated. The newly formed bone rarely proliferated into the central 5-mm defect area at 4 weeks (Figure 2 and Figure 3). The respective values of MD and mineralized tissue volume (MV) in VOI_3 represented the agglomerate of newly formed bone and residual non resorbed material, whereas the MD and MV in VOI_2 represented the osteoclast induced resorption of the materials at 4 weeks (Table 3 and Table 4).

The initial MD of the materials at week 0 was 670.69 ± 8.66 mgHA/ccm in DBBM, 803.84 ± 10.38 mgHA/ccm in α-TCP + DBBM, 795.16 ± 6.05 mgHA/ccm in BBCP3%HA, and 812.62 ± 28.57 mgHA/ccm in BBCP_6, respectively. Compared to the MD of VOI_1 after 4 weeks, all materials, except DMMB, showed reduced values. In the central area (VOI_2) all combination materials, including α-TCP + DBBM, BBCP_3 and BBCP_6 groups, showed significantly lower MD at week 4 when compared to 0 week 0 (*p* = 0.0083 in α-TCP + DBBM, *p* = 0.0050 in BBCP_3, *p* = 0.0191 in BBCP_6, respectively), while DBBM maintained similar MD values at weeks 0 and 4 (Table 3). Importantly, significantly lower MD values of DBBM were observed when compared with the other synthetic combined material groups at weeks 0 and 4. Despite the loss of MD observed in the combinations, there were no statistically significant differences in residual MV, as well as in MV/TV in the central 5-mm defect area (VOI_2), between any material groups at 4 weeks, corresponding to the histological findings.

The comparison of the MD values at week 0 with those of week 4 in the peripheral defect areas (VOI_3) revealed a decrease for all groups, except for the DBBM that yielded an obvious increase. On the other hand, no significant differences were observed in the MV and MV/TV between groups in peripheral 2.5-mm defects at 4 weeks (VOI_3; Table 4).

## 3. Discussion

The long-term success of bone augmentation procedures depends on the volume maintenance capacity of the bone substitute applied, the latter being critically dependent on its degradation kinetics. Hence, too slow degradation decreases the amount of newly formed bone [11]. As it is known for its long-term volume maintenance, DBBM was applied in combination with autogenous bone [3]. The BBCPs used in the present study were developed to fine-tune α-TCP degradation. The aim of the present study was to assess the effect of a combined use of a DBBM with a rapidly degrading α-TCP and two α-TCP based newly developed BBCPs on biomaterial degradation and new bone formation. As there were no previous data available, a 1:1 ratio was chosen arbitrarily. A critical-size calvaria defect model was used to study the early bone forming capacity of the combined biomaterials at 4 weeks after surgery. Both histological preparations, as well as micro-CT scans, were assessed for potential differences in bone formation and resorption on the DBBM combinations applied. Histologically, all tested biomaterials were osteoconductive. New mineralized bone volume slightly increased when DBBM was applied in combination with α-TCP or BBCP 3%HA/97% α-TCP, or BBCP 6%HA/94% α-TCP (Table 2). The absence of a statistically significant difference, however, did not refute the null hypothesis. Nonetheless, DBBM alone showed significantly lower volumes of new bone compared to the NC group.

Micro-CT analysis revealed no significant differences in MV, MV/TV and HDC between any groups, neither when analyzed for the whole defects nor for the peripheral or central regions of the defects. The MD measurements in the center of the defects, however, showed significant differences among the groups. Mineral density of the combinations measured at week 0 significantly decreased when compared to week 4 in contrast to the DBBM alone, the latter showing similar MDs at both time points. The analysis of the effect of the biomimetic HA coating of α-TCP revealed no significant differences on its resorption level when applied in combination with DBBM. The mineralized tissue volume was, thus, maintained, despite ongoing resorption of all the combination products in situ after 4 weeks.

The effect of a biomimetic HA coating for α-TCP on resorption and concomitant new bone formation has been previously presented using the same calvaria model [13]. Three different compounds, namely, 3%HA/97%α-TCP, 12%HA/88%α-TCP and 23%HA/77%α-TCP, were tested for the parameters of new bone formation at 3 weeks and at 3 months. After 3 weeks, no significant differences between the BBCPs with different levels of HA coating could be observed [13]. In contrast, samples with BBCP_3 showed significantly less residual material and promoted more new bone volume as compared to the other groups at 3 months. When compared to the previous study [13], the addition of DBBM appeared not to influence the kinetics of the BBCP_3 resorption and bone formation volume within the first weeks after augmentation. It might be assumed that the effect of DBBM, as a very slow resorbing and, thus, volume maintaining bone substitute, would become evident at a later time point, most likely after the complete resorption of the BBCP_3 within the remodeling phase. Nevertheless, it has to be emphasized that mineral density and histological data reminiscent of creeping substitution indicated that within the first 4 weeks, the combination products experienced some resorption in comparison to DBBM alone. Despite this resorption activity, the combination products maintained the volume inside the defects similar to DBBM alone. Hence, the addition of α-TCP or BBCP 3%HA/97% α-TCP or BBCP 6%HA/94% α-TCP to DBBM in a 1:1 ratio did not jeopardize their early volume maintenance capacity, despite comprising a reduced amount of DBBM.

In the central 5-mm defect area at 0 versus 4 weeks, it was found that α-TCP, BBCP_3 and BBCP_6 absorbed to the same level regardless of their HA coating (Table 3). In addition, the defects filled with BBCP_3 or BBCP_6 yielded very similar bone formation volumes at 4 weeks to those obtained for DBBM + α-TCP. Thus, the biomimetic coverage of the α-TCP with hydroxyapatite, expected to prolong the degradation of α-TCP granules, did not have a major impact on resorption levels or bone neo-formation during early healing. These findings were in line with the results of the study by Kunert-Keil et al., comparatively analyzing β-TCP and the respective derived biphasic calcium phosphate in a rat calvaria defect model after 4 weeks [18]. New bone formation did not differ between the two differently augmented defects; however, significantly higher mRNA levels of the bone resorption marker Acp5 and the osteogenic differentiation marker Runx2 was detected in the β-TCP group as opposed to the biphasic calcium phosphate. The histological evaluation of bone at early time points in that study was obviously not sufficient to detect differences in bone formation comparing pure TCP-based substitutes and their respective biphasic counterparts.

The reduced new bone formation observed after 4 weeks in the DBBM-group compared to the non-augmented control group was in line with previous published data [13]. Using a porcine calvaria defect model, Titsinides et al. reported the reduced bone formation in a case of a defect filled with DBBM or β-TCP after 8 and 12 weeks [19]. New bone formed at the expense of β-TCP, which was found to be in the process of degradation, while DBBM remained almost intact. These findings were in agreement with a limited DBBM resorption previously reported by other authors [14,15]. However, the data on long-term stability and degradation kinetics in pre-clinical settings are still missing. Given that the combination of DBBM with α-TCP or BBCP 3%HA/97% α-TCP or BBCP 6%HA/94% α-TCP resulted in a slight increase of new bone volume when compared to DBBM alone, the α-TCP based synthetic materials might possibly achieve a better balance between resorption of the biomaterial and new bone formation. In this respect, the addition of α-TCP based materials to DBBM may provide two benefits: counteracting the DBBM-caused delayed bone formation and opening more space for new bone formation upon resorption.

There were several limitations inherent in the present study. Although the critical-sized calvaria defect model of rabbits is commonly applied in the study of bone formation, this model cannot completely mimic the clinical situation of the GBR techniques, due to the limited space available. Hence, other models forming bone beyond the skeletal envelope may shed more light on the regenerative capacity of these biomaterials [20]. Only one time point and one ratio of DBBM + α-TCP, BBCP 3%HA/97%α-TCP and BBCP 6%HA/94%α-TCP were tested. To fully elucidate the effect of tested combinations on the performance of DBBM, preferably 3- and 6-months data need to be generated. Moreover, it may be possible that various ratios of the two components may depict more clearly the effect of the addition of synthetic compounds to DBBM. The tested sample size (*n* = 6) was calculated by power analysis before starting the experiments, but the sample size had to be decreased due to a severed dura mater during surgery. In summary, the effect of combination of the α-TCP based bone substitute materials with the DBBM on de novo bone formation was minor in the chosen experimental settings. Similar effects were observed for the different levels of biomimetic HA coating of α-TCP on its resorption.

## 4. Materials & Methods

### 4.1. Materials

The tested materials were provided by Geistlich Pharma AG, Wolhusen, Switzerland. Four different substitutes: (1) NC (empty), (2) DBBM alone, (3) α-TCP + DBBM (1:1), (4) 3%HA/97%α-TCP + DBBM (1:1) (BBCP_3), and (5) 6%HA/94%α-TCP + DBBM (1:1) (BBCP_6) were investigated (Figure 1).

### 4.2. Animals

Nineteen New Zealand White female rabbits, approximately 16 weeks of age (3.0–3.4 kg), were used in the present study. During the acclimatization period and throughout the experiment, the animals were housed in the Central Animal Care Facility at the University of Berne (temperature 19–21 °C, humidity 45% ± 10%, a light/dark cycle of 12:12 h). The animals were housed without excessive or disturbing noises and fed with a standard diet and water *ad libitum*. The study considered the NC3Rs, UK guidelines, and is reported according to the ARRIVE guidelines for preclinical in vivo studies. The study was submitted to and approved by the Committee for Animal Research, Canton of Berne, Switzerland (Nr: BE 89/17). Thirty critical size defects were used for the calvarial surgeries, and the 8 intact parietal bones discs were harvested and used as a reference.

### 4.3. Anesthesia

The animals were premedicated subcutaneously (s.c.) with methadone (0.3 mg/kg), dexmedetomidine (100 g/kg) mixed with ketamine 15 mg/kg (Narketan^®^, Vetoquinol AG, Bern, Switzerland). After reaching an appropriate depth of sedation, the eyes of the animals were lubricated (Bepanthen^®^ Augen- und Nasensalbe, Bayer Vital GmbH, Leverkusen, Germany) and pure oxygen was administered through a facemask. An intravenous (i.v.) catheter was inserted in one of the marginal auricular veins. Ropivacaine 0.75% was locally administered on the surgery site. General anesthesia was maintained with isoflurane (Forene^®^, Abbvie AG, Baar, Switzerland) vaporized in pure oxygen through a Jackson Rees modified T-piece breathing system targeting a maximal Et Iso of 1–1.3%.

### 4.4. Surgical Procedures

The skin of the rabbit was incised from the nasal bone to the mid-sagittal crest, and the periosteum was elevated to expose the parietal bone. Two critical-size 10-mm diameter calvaria bone defects were prepared with a trephine under copious irrigation with sterile saline. Maximal care was taken to avoid injury to the *dura mater*. Allocation of the 5 applied treatment modalities, (1) NC, (2) DBBM, (3) α-TCP + DBBM, (4) BBCP_3 and (5) BBCP_6 (*n* = 6, each) was randomized according to the systematic random protocol (www.randomization.com (accessed on 17 January 2019)). The 600 μL of blood were collected from the auricular artery per animal. The 300 μL of blood were used to mix with each granule and implanted into a defect, and the 300 μL filled up into the NC. After implantation of the materials, the 12.5 mm × 13.0 mm-sized resorbable collagen barrier membrane (BioGide^®^, Geistlich Pharma AG, Wolhusen, Switzerland) was used to cover the defect sites. The wound was closed in two layers with interrupted sutures using 4–0 Vicryl^®^ and 4–0 Monocryl^®^ sutures (Ethicon, Somerville, NJ, USA), respectively. Wound surfaces were further sealed with a spray film dressing (OPSITE^®^ SPRAY, Smith & Nephew, London, UK).

### 4.5. Postoperative Procedures

The rabbits were left to recover under infrared lights and administration of oxygen following surgery. Peri-operative antimicrobial prophylaxis (procaine penicillin 150,000 IU/mL + benzathine penicillin 150,000 IU/mL; 0.01 mL/kg s.c, Duplocillin^®^, MSD Animal Health, Luzern, Switzerland) were applied. Postoperative analgesia consisted of meloxicam (Metacam^®^, Boehringer Ingelheim, Ingelheim, Germany) 0.5 mg/kg i.v. administered after surgery and repeated once daily for 4 days. Regular monitoring included assessment of water and food consumption and assessment of pain at regular intervals (composite pain scale and grimace scale). If indicated, Buprenorphine (Temgesic^®^, Rechitt Benckiser, Wallisellen, Switzerland) was administered at 20 g/kg s.c. every 8 h during the postoperative 3 days. The animals were sacrificed after a healing period of 4 weeks with an overdose of pentobarbital 120 mg/kg i.v. (Streuli Pharma AG, Uznach, Switzerland) following the premedication with ketamine 65 mg/kg and xylazine 4 mg/kg s.c. in the neck area.

### 4.6. Micro-CT Analysis

The collected calvaria specimens were fixed in 10% neutral formalin for 7 days at room temperature and replaced in 70% ethanol at 4 °C. The specimens were then subjected to micro-CT scans using a desktop cone beam scanner (μCT 40, ScancoMedical AG, Brüttisellen, Switzerland). The X-ray source was set at 70 kV with 114 μA. An isotropic voxel size of 18 μm showed an image matrix of 2048 Å~2048 pixels. The micro-CT images were then analyzed and reconstructed by using 3D structural analysis software (Amira, Visualization Sciences Group, Düsseldorf, Germany). The primary volume of interest (VOI_1) were 10-mm diameter, full thickness cylinders, selected corresponding to the dimensions of the defect sites (Supplementary Figure S1). Furthermore, a central 5-mm diameter full thickness cylinder in the defect sites as the secondary VOI (VOI_2), and the peripheral 2.5-mm diameter full thickness cylinder in the defect sites as the third VOI (VOI_3) were chosen. The parameters included MV (mm^3^), MV/TV, %), MD (mgHA/ccm, initial material at week 0 and, in the defect site, at week 4). Moreover, HDC (relative % to whole defect length; 10 mm) weas calculated by two experienced examiners who were blinded to the treatment modalities. The intact calvarial bone (intact bone, *n* = 8) was also evaluated for reference.

### 4.7. Histological Processing and Histomorphometric Analysis

All specimens were trimmed, dehydrated in ascending concentrations of ethanol, and embedded in methyl methacrylate without decalcification. The embedded tissue blocks were cut sagittally in the middle of the defects at approximately 1000-μm thick ground sections using a slow-speed diamond saw (VC-50; LECO, St. Joseph, MI, USA). After mounting on acrylic glass slabs, the sections were ground and polished to a final thickness of 200 μm (Knuth Rotor-3; Struers, Ballerup, Denmark). The sections were stained with toluidine blue combined with fuchsin and the images photographed under a digital microscope (VHX-6000, Keyence, Osaka, Japan). Morphometric analysis was performed by a graphic software (Photoshop CC; Adobe, San Jose, CA, USA) using the corresponding 10-mm initial defect area as the region of interest (ROI). The parameters included new bone area (NBA, %), bone marrow area (BMA, %), connective tissue area (CTA, %), total residual material area (RMA, %), residual DBBM material area (RMA-D, %), residual synthetic material area (RMA-S, %), and HDC (%). These were calculated as a relative % to the total augmentation area (mm^2^) by two experienced examiners, while blinded to the treatment groups.

### 4.8. Statistical Analysis

The data represented the means and standard deviations (SD) for all quantitative data in the tables. The statistical analysis was performed by one-way analysis of variance (ANOVA) with Tukey test by using a statistical program (GraphPad Prism X9 software: GraphPad Software, Inc., La Jolla, CA, USA). The level of significance was set at α = 0.05.

## 5. Conclusions

Within the limits of this 4-weeks preclinical in vivo study, the addition of the α-TCP based bone substitutes to DBBM tended to improve new bone formation when compared to DBBM alone. Very limited resorption of the α-TCP based bone substitutes independent of their HA content, was observed indicating similar levels of osteoconductivity of the combination products compared to the DBBM alone. In conclusion, the impact of the combination, as well as differences of α-TCP based material components, in the bone augmentation potential was minor. Further preclinical studies with a larger sample size and long-term healing periods will be necessary to elucidate the potential benefits of the combination biomaterials and clarify the relationship between material resorption and bone neoformation.

## Figures and Tables

**Table 1:** Quantified micro-CT values in a 10-mm defect VOI (VOI_1).

Parameter	Group 1	Group 2	Group 3	Group 4	Group 5	Reference
	NC (*n* = 6)	DBBM (*n* = 6)	α-TCP + DBBM (*n* = 6)	3%HA/ α-TCP + DBBM (*n* = 6)	6%HA/ α-TCP + DBBM (*n* = 5)	Intact Bone (*n* = 8)
MV (mm^3^)	25.43 ± 7.73	59.31 ± 11.42	68.93 ± 16.17	64.30 ± 17.83	71.30 ± 9.62	71.53 ± 8.59
		vs. NC **	vs. NC #	vs. NC #	vs. NC #	
MV/TV (%)	11.50 ± 1.71	29.06 ± 3.51	32.18 ± 6.43	29.83 ± 7.57	31.28 ± 2.88	35.94 ± 7.64
		vs. NC #	vs. NC #	vs. NC #	vs. NC #	
MD (mgHA/ccm)	611.74 ± 18.66	689.05 ± 17.95	728.08 ± 27.65	716.13 ± 22.54	724.69 ± 18.26	680.37 ± 13.48
		vs. NC #	vs. NC #	vs. NC #	vs. NC #	
			vs. DBBM *			
HDC (%)	48.40 ± 27.37	25.80 ± 9.85	33.48 ± 5.83	32.33 ± 11.98	39.66 ± 7.24	

Mean values with standard deviations. * statistically significant difference denotated by * at *p* < 0.05, ** at *p* < 0.01 and # at *p* < 0.001. MV; mineralized tissue volume, TV; total volume, MD; mineral density, HDC; horizontal defect closure. The data of the intact calvarial bone without surgery is shown as a reference.

**Table 2:** The histomorphometry in 10-mm defect ROI.

Parameter	Group 1	Group 2	Group 3	Group 4	Group 5	Reference
	NC (*n* = 6)	DBBM (*n* = 6)	α-TCP + DBBM (*n* = 6)	3%HA/ α-TCP + DBBM (*n* = 6)	6%HA/ α-TCP + DBBM (*n* = 5)	Intact Bone (*n* = 8)
NBA (%)	18.82 ± 5.36	8.94 ± 3.38	12.74 ± 4.76	13.25 ± 7.77	13.62 ± 3.41	52.35 ± 6.32
		vs. NC *				
BMA (%)	3.99 ± 1.51	1.72 ± 1.08	2.91 ± 1.77	2.37 ± 2.16	3.36 ± 0.67	38.02 ± 7.64
CTA (%)	77.19 ± 6.78	63.66 ± 3.90	60.97 ± 3.63	62.33 ± 7.21	59.53 ± 3.16	9.63 ± 2.59
RMA (%)		25.68 ± 2.38	23.38 ± 3.50	22.05 ± 4.58	23.49 ± 5.98	
RMA-D (%)		25.68 ± 2.38	16.15 ± 2.27	18.26 ± 4.46	18.25 ± 4.55	
			vs. DBBM #	vs. DBBM **	vs. DBBM *	
RMA-S (%)			7.22 ± 2.41	3.79 ± 1.71	5.24 ± 3.26	
HDC (%)	52.66 ± 27.30	31.85 ± 13.66	51.19 ± 30.99	54.14 ± 21.14	51.62 ± 20.64	

Mean values with standard deviations. Statistically significant difference denoted by * at *p <* 0.05), ** at *p* < 0.01 and # at *p* < 0.001. NBA; new bone area, BMA; bone marrow area, CTA; connective tissue area, RMA; residual material area, RMA-D; residual DBBM material area, RMA-S; residual synthetic material area, HDC; horizontal defect closure. The data of the intact calvarial bone without surgery is shown as a reference.

**Table 3:** Quantified micro-CT values in a central 5-mm defect VOI (VOI_2).

Parameter (Central 5 mm)	Group 2	Group 3	Group 4	Group 5
	DBBM (*n* = 6)	α-TCP + DBBM (*n* = 6)	3%HA/ α-TCP + DBBM (*n* = 6)	6%HA/ α-TCP + DBBM (*n* = 5)
MV (mm^3^)	9.55 ± 3.25	12.77 ± 4.94	10.27 ± 3.21	11.04 ± 3.08
MV/TV (%)	18.57 ± 4.47	24.03 ± 9.36	18.93 ± 5.25	19.34 ± 4.99
		vs. DBBM #	vs. DBBM #	vs. DBBM #
MD (mgHA/ccm)	666.13 ± 21.76	735.48 ± 47.90	722.77 ± 26.96	748.83 ± 23.59
		vs. DBBM **	vs. DBBM *	vs. DBBM **

Mean values with standard deviations. Statistically significant difference denoted by * at *p <* 0.05, ** at *p* < 0.01 and # at *p* < 0.001. MV; mineralized tissue volume, TV; total volume, MD; mineral density.

**Table 4:** Quantified micro-CT values in a peripheral 2.5-mm defect VOI (VOI_3).

Parameter (Peripheral 2.5-mm)	Group 2	Group 3	Group 4	Group 5
	DBBM (*n* = 6)	α-TCP + DBBM (*n* = 6)	3%HA/ α-TCP + DBBM (*n* = 6)	6%HA/ α-TCP + DBBM (*n* = 5)
MV (mm^3^)	49.76 ± 9.31	56.16 ± 13.69	54.03 ± 14.98	60.26 ± 6.91
MV/TV (%)	32.56 ± 4.35	34.90 ± 7.06	33.46 ± 8.59	35.26 ± 2.21
MD (mgHA/ccm)	692.76 ± 18.94	724.74 ± 23.15	711.32 ± 23.31	720.29 ± 17.85

Mean values with standard deviations. MV; mineralized tissue volume, TV; total volume, MD; mineral density.

## Data Availability

The data presented in this study are available on request from the corresponding author.

## Supplementary Materials

The supporting information can be downloaded at: https://www.mdpi.com/article/10.3390/ijms231810516/s1.
