# Supplementary figures and images for: Addition of Synthetic Biomaterials to Deproteinized Bovine Bone Mineral (DBBM) for Bone Augmentation—A Preclinical In Vivo Study
Source: Int J Mol Sci. 2022 Sep 10;23(18):10516. doi: 10.3390/ijms231810516 (PMC9505841; doi:10.3390/ijms231810516)

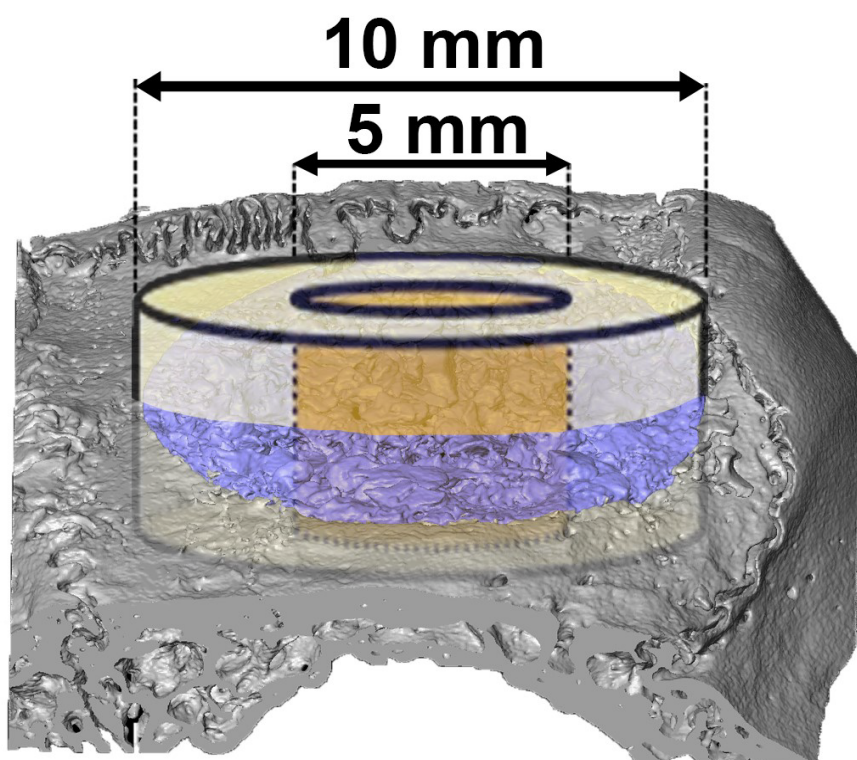

VOI\_1

VOI\_2

VOI\_3

Supplement: Supplementary file 1 [file ijms-23-10516-s001.zip › ijms-1869732-supplementary.pdf]
